# The Feeding of Girls at Schools

**Published:** 1893-01-14

**Authors:** 


The Hospital

An Institutional Journal of

Science, Medicine, Nursing, and Philanthropy.

*For Hospitals, Asylums, Infirmaries, Dispensaries, Medical Practitioners, Students, Nurses, and the Charitable Public.*

Vol. XIII.—No. 329.] [Jan 14, 1893.

### The Feeding of Girls at Schools.

Some few weeks ago we published an article on "The Public Schoolboy's Body." The attention thus directed to the subject was productive of good results in the right quarters. But if we may believe the representations since made to us by anxious parents, there are far worse evils to be found in girls' schools than have ever been discovered in those where boys are domiciled. A judicious, and not over-anxious mother informed the present writer that her three boys, after being sent from home to school, all improved decidedly in health, in consequence of the vigorous exercise and the abundance of plain, but well-cooked food, which were the marked features of their school life. Of the same mother's four girls, two were sent to a school where the food was excellent and abundant, and two others to a different school, where the meals were but three a day, and of a poor and unappetising quality. The two former girls, like their brothers, improved in constitution and health during their school life, the two latter—as their mother expressed it—returned home with constitutions ruined. All those boys and girls were children of the same parents, and were all in good health and of sound constitution when sent to school.

That is a single case. Without being looked for, other similar cases have been reported to the writer in sufficient number to justify the conclusion that widespread injury of a lifelong kind is being inflicted upon young girls at large numbers of English boarding schools. The injury is inflicted upon those who have neither the intelligence nor the power to protect themselves. Many young girls are quite ignorant of the way in which they ought to be fed, and many more are indifferent. They have poor appetites, and feeble health, and they take what is set before them without thinking of complaint.

There are several aspects of this question which appeal to the medical physiologist. If he be a parent he is impressed by the great unhappiness which springs from the feeble health engendered by insufficient and unappetising food. The young girl, like all young creatures, ought to be happy, full of brightness, cheeriness, and fun. If she is not happy there is something radically wrong, and it ought to be found out. But the injuries and miseries which the parent sees are as nothing compared with the distressing and life-long train of indescribable evils which the medical man pictures to himself as the portion of those unhappy girl-children whose health has been effectually and for ever destroyed by their school life.

Will parents consider with us this important family question? And, first, let it be pointed out that the differences between girls and boys are important and manifold. The boy is of a rough mould, and full of self-assertion; the girl inclines more to be timid, and to make few demands for herself. The boy is made for aggression, and has his own ways of making good his claims to due consideration; the girl can hardly be aggressive if she would; she leans upon what is stronger than herself, and if it bends she must needs bend with it. This radical difference between boys and girls ought surely to be taken into account. In these scientific days every public and private question is being reconsidered; it is brought to the bar of the knowledge and science of the times; and that is how it should be. What is the use of our labours in gaining physiological and other scientific knowledge if it is not to serve the practical purpose of making us healthier, happier, and more serviceable men and women? Our grandfathers and grandmothers were excellent people no doubt; but they were not physiologists, and in the matter of the care and culture of the bodies of ourselves and our children they are the very last people in the world whose examples and precepts we should think of making patterns for the present generation.

The girl-child is a rare and delicate flower, and she requires the best skill of the wisest physiological gardener to bring her to perfection. In the management of such a flower there are four absolutely indispensible conditions: she must have abundance of fresh pure air: abundance of appetising food and sleep; abundance of joyful play; and abundance of moderation in work both for body and mind. The growing period is not the time for work, it is the time for growth. If you insist upon a great deal of work in youth, it is as if you should make frantic efforts to drag the ripe fruit out of the trunk and branches of the tree in the flowering season. If your plant be a wall-fruit, you may gently train up its branches in the direction of the sun, and you may apply stimulating nourishment to its roots. But fruit of a character which is ripe and fit for eating is not to be even so much as looked for until the latest and autumn stage of summer development.

This illustration almost exactly expresses the teaching of science with regard to the rearing of young girls. Our present object is to insist and re-insist that they are to be abundantly and appetisingly fed, and we must keep to this point. But we cannot help making a most earnest protest, by the way, against the long hours and health-ruining studies to which so many young girls are, unhappily, driven by the folly of the times.

The one thing which is the most important of all for a girl between the age of nine and nineteen is the feeding of her body. That is the period in which, like a beautiful temple, the constitution of the future woman is being built. The physiologist can see the process of cell being added to cell, and structure rising and being completed not only year by year, and month by month, but day by day, hour by hour, moment by moment. Unfortunately too, he can, in many cases, see this structure built up one day, torn down the next, and finally scattered in the hopeless ruin of premature consumption and death. Is it not a man's clear duty who sees these things to speak of them, and to speak so that the world shall at least hear, whether it heeds or not! What need a Sir Crichton Browne care though high school mistresses affect to laugh, and newspaper writers ignorant of physiology, indulge in gentle jests? The physiologist, like other men of science, speaks the truth as Nature herself has irresistibly compelled him to see it. If the world will not hear, so much the worse for the world. It must hear, or it alone must bear the penalty of its deafness.

In the feeding of schoolgirls the first thing to do is to provide them with an appetite; and this can always be done if intelligence is at the head of affairs. The girls should have a liberal supply of fresh, well-cooked meat at least twice a day. In addition to this they should have eggs and fish three or four days a week. A great variety of thoroughly nourishing puddings should be provided for them; and plenty of vegetables of the freshest and the best. Milk should be abundant, and fresh fruit plentiful. They should have four good meals in the day. They should never study within an hour and a half after one meal, and half an hour before the next. Four hours a day of thoroughly diligent study is as much as the average girl can well stand. Five hours are too long for many girls, and six too much for all but the very strongest. We have purposely avoided the laying down of minute rules; but we hope that enough has been said to show parents what ought to be done for their daughters during school life, and to persuade them to insist that it shall be done.

